# Laparoscopic versus open right posterior sectionectomy: an international, multicenter, propensity score-matched evaluation

**DOI:** 10.1007/s00464-020-08109-y

**Published:** 2020-11-02

**Authors:** Nicky van der Heijde, Francesca Ratti, Luca Aldrighetti, Andrea Benedetti Cacciaguerra, Mehmet F. Can, Mathieu D’Hondt, Fabrizio Di Benedetto, Arpad Ivanecz, Paolo Magistri, Krishna Menon, Michail Papoulas, Marco Vivarelli, Marc G. Besselink, Mohammed Abu Hilal

**Affiliations:** 1 Department of Surgery, Southampton University Hospital, Southampton, UK; 2 Department of Surgery, Cancer Centre Amsterdam, Amsterdam UMC, University of Amsterdam, Amsterdam, the Netherlands; 3 Department of Surgery, San Raffaele Hospital, Milan, Italy; 4 HPB Surgery and Transplantation Unit, Department of Clinical and Experimental Medicine, Polytechnic University of Marche, Ancona, Italy; 5 Department of Surgery, Fondazione Poliambulanza - Instituto Ospedaliero, Brescia, Italy; 6 Department of Surgery, Lokman Hekim University School of Medicine, Ankara, Turkey; 7 Department of Surgery, AZ Groeninge Hospital, Kortrijk, Belgium; 8 Department of Surgery, University of Modena, Modena, Italy; 9 Department of Abdominal and General Surgery, University Medical Centre Maribor, Maribor, Slovenia; 10 Department of Surgery, King’s College Hospital, London, UK

**Keywords:** Laparoscopic surgery, Liver surgery, Minimally invasive surgery, Operative outcomes, Propensity score matching, Surgical procedure

## Abstract

**Background:**

Although laparoscopic liver resection has become the standard for minor resections, evidence is lacking for more complex resections such as the right posterior sectionectomy (RPS). We aimed to compare surgical outcomes between laparoscopic (LRPS) and open right posterior sectionectomy (ORPS).

**Methods:**

An international multicenter retrospective study comparing patients undergoing LRPS or ORPS (January 2007—December 2018) was performed. Patients were matched based on propensity scores in a 1:1 ratio. Primary endpoint was major complication rate defined as Accordion ≥ 3 grade. Secondary endpoints included blood loss, length of hospital stay (LOS) and resection status. A sensitivity analysis was done excluding the first 10 LRPS patients of each center to correct for the learning curve. Additionally, possible risk factors were explored for operative time, blood loss and LOS.

**Results:**

Overall, 399 patients were included from 9 centers from 6 European countries of which 150 LRPS could be matched to 150 ORPS. LRPS was associated with a shorter operative time [235 (195–285) vs. 247 min (195–315) *p* = 0.004], less blood loss [260 (188–400) vs. 400 mL (280–550) *p* = 0.009] and a shorter LOS [5 (4–7) vs. 8 days (6–10), *p* = 0.002]. Major complication rate [*n* = 8 (5.3%) vs. *n* = 9 (6.0%) *p* = 1.00] and R0 resection rate [144 (96.0%) vs. 141 (94.0%), *p* = 0.607] did not differ between LRPS and ORPS, respectively. The sensitivity analysis showed similar findings in the previous mentioned outcomes. In multivariable regression analysis blood loss was significantly associated with the open approach, higher ASA classification and malignancy as diagnosis. For LOS this was the open approach and a malignancy.

**Conclusion:**

This international multicenter propensity score-matched study showed an advantage in favor of LRPS in selected patients as compared to ORPS in terms of operative time, blood loss and LOS without differences in major complications and R0 resection rate.

**Electronic supplementary material:**

The online version of this article (10.1007/s00464-020-08109-y) contains supplementary material, which is available to authorized users.

Laparoscopic liver surgery (LLS) has evolved greatly in the past few decades. The first laparoscopic liver wedge resection was reported in 1991, [1] after which the first anatomic partial hepatectomy followed in 1996 [2]. Nowadays, LLS includes both minor and major liver resections, as well as hepatectomies for living liver donation. Benefit of LLS mostly includes less postoperative complications, less blood loss and a shorter length of hospital stay [3–5]. In high-volume centers, the use of LLS has increased exponentially in recent years. For instance, between 2000 and 2015, the percentage of LLS in four large European centers grew from 5 to 43% [6].

In 2014, a surgical difficulty score was developed for LLS [7]. The right posterior sectionectomy (RPS) is classified as a procedure with high difficulty scores due to its technical complexity. This is explained by the time consuming parenchymal transection and the close proximity to the hepatic veins and its branches, with risk of massive bleeding. Laparoscopic RPS (LRPS) scores nine out of 10 points in the ‘high difficulty’ group. High difficulty LLS cases were defined as: cases that should be handled by more experienced surgeons who regularly perform intermediate difficulty LLS and have performed ≥ 50 LLS cases [7].

LRPS is not performed on a regular bases and most studies either included a small sample size (between 18 to 61 procedures) or were single center studies [8–11]. These studies concluded that although it seemed feasible and safe to perform LRPS, it was emphasized that studies with larger number of patients are necessary.

The aim of this study was to compare perioperative outcomes between LRPS and open right posterior sectionectomy (ORPS) for all indications using propensity score matching. Additionally, risk factors were explored for postoperative outcomes that were significantly different between the two approaches.

## Materials and methods

### Design and study population

This was a multicenter retrospective analysis comparing all consecutive patients of 9 different European centers (Amsterdam UMC, Amsterdam, The Netherlands; Southampton University Hospital, Southampton, United Kingdom; King’s College, London, United Kingdom; San Raffaele Hospital, Milan, Italy; University of Modena and Reggio Emilia, Modena, Italy; Polytechnic University of Marche, Ancona, Italy; Groeninge Hospital, Kortrijk, Belgium; Lokman Hekim Hospital, Ankara, Turkey; University Medical Centre Maribor, Maribor, Slovenia). All patients of 18 years and older undergoing LRPS and ORPS for all indications between January 2007 and December 2018 were included. According to the Health Research Authority in the UK, Research Ethics Committee and Health Research Authority approval is not required for research databases; this includes the release of non-identifiable data for analysis. Owing to the retrospective nature of the study, written informed consent was not obtained [12]. This study is reported in accordance with the STROBE statement [13].

Exclusion criteria were patients undergoing multivisceral resections or synchronous procedures and patients undergoing resections of more than 3 segments of the liver. A cholecystectomy during the same procedure was permitted.

A short survey of seven questions was conducted (on volume of laparoscopic liver surgery, surgical technique and the use of training) to obtain background information of each center. All patients underwent preoperative evaluation that included physical examination, blood tests, either a MRI or CT scan. All patients were discussed in a multidisciplinary team meeting prior to start of treatment. Surgery was performed in accordance with local standard practice and there was no set surgical technique. No specific selection criteria were used to allocate patients to open or laparoscopic surgery. The applied surgical modality was based on surgeon choice and there were no specific contra-indications for laparoscopic surgery. Postoperative care was in accordance with each unit’s standard practice, all hospitals used the enhanced recovery after surgery (ERAS) protocol. There were no set criteria to decide when patients were ready to be discharged home.

### Outcomes

Baseline characteristics and short term outcomes were collected by the local investigators for all patients. Laparoscopic cases converted to open were included in the laparoscopic group in order to perform an intention-to-treat analysis. The primary endpoint of this study was major complication rate. Secondary endpoints were perioperative outcomes as estimated blood loss, operative time, length of stay, R0 resection rate and 90-day mortality.

### Definitions

Segmental liver anatomy was reported according to the Brisbane classification and segments VI and VII were considered as right posterior [14]. Comorbidities were defined using the ASA classification. The Accordion grading system and the comprehensive complication index were used for postoperative morbidity [15, 16]. An Accordion grade ≥ 3 was considered a major complication. Severity of cirrhosis was defined with the Child–Pugh score [17]. Surgical margin was defined as R1 (non-radical margin) whenever the width was microscopically < 1 mm from the resection margin. Tumor size was reported as the diameter of the largest lesion if multiple lesions were present.

### Statistical analysis

Non-normally distributed data were expressed as median with interquartile range (IQR) and normally distributed data as mean and standard deviation (SD). In the unmatched cohort, demographic and clinical characteristics were compared using χ^2^ test or Fishers exact test for categorical variables, Mann–Whitney U test for non-normally distributed continuous variables and *t* test for normally distributed continuous variables. In the matched cohort, continuous data were compared using paired t-test, binary variables using McNemar test and for categorical or non-normally distributed continuous variables the Wilcoxon signed rank test. A sensitivity analysis was performed excluding the first 10 laparoscopic right posterior sectionectomies of each center to correct for the learning curve.

Regression analysis was carried out to identify risk factors for outcomes that were statistically significantly different between the laparoscopic and open approach; variables with a *p* value < 0.2 in univariable analysis were subsequently entered in a multivariable regression analysis. *p* values of < 0.05 were considered statistically significant. Data were analyzed using IBM SPSS version 25.0 (SPSS Inc., Chicago, IL, USA). Propensity score matching (PSM) was performed using R software (R Core Team, R foundation for Statistical Computing, Vienna, Austria).

### Propensity score matching

A propensity score (PS) was calculated to reduce the effects of potential confounding between groups. Patients were matched based on propensity scores in a 1:1 ratio. Variables selected for matching to compare the groups were: age (≤ 75 years, > 75 years), sex, ASA classification (ASA 1–2 or ASA 3–4), neoadjuvant chemotherapy, previous abdominal surgery, previous liver surgery, indication for surgery (benign or malignant), cirrhosis (yes or no) and number of lesions (one or multiple). The variables included for matching were based on previous literature. Nearest-neighbor matching without replacement within a caliper was used. The size was set as 0.2 of the standard deviation of the logit of the estimated propensity score according to the suggestion of Austin [18]. Patients who were found to be outside the caliper or patients who were unmatched, were excluded. Standardized mean differences (SMD) were calculated to assess if the matches were well balanced, a SMD < 0.1 is considered well balanced.

## Results

### Background information centers

Participating centers started with LLS between 2003 and 2014 (median 2009). Seven out of nine centers (77.7%) had undergone specific laparoscopic liver training prior to starting performing laparoscopic liver surgery. Three centers (33.3%) were training other surgeons in LLS. None of the centers had performed a LRPS before the inclusion period. The first LRPS was done between 2009 and 2016 (median 2012). Number of laparoscopic liver resections performed during the 12-year inclusion period was a median of 470 cases (184–780 IQR). Four centers (44.4%) most commonly used ultrasound guided resection for RPS without vascular control and the resection line was delineated 5–10 mm from the right hepatic vein. Three centers (33.3%) preferred inflow control after hilar dissection and identification of the RPS vessels. The resection was guided by the ischemia line and intraoperative ultrasound. Two centers (22.2%) stated not to have a specific preference, they decided their technique based on the location of the tumor.

### Baseline characteristics

Overall, 399 patients after RPS could be included for analysis, 171 in the LRPS group and 228 patients in the ORPS group. Baseline characteristics are summarized in Table 1. There were statistically significant imbalances between the two groups for sex and number of lesions. There was a trend toward more ASA 3–4 patients in the open group (*p* = 0.07). In the first half of the inclusion period, between 2007 and 2013, 27 of the laparoscopic cases (15.8%) were operated compared to 132 of the open cases (57.9%).

**Table 1:** Baseline characteristics before and after propensity score matching

	Before PS matching			After PS matching			
	Laparoscopic RPS (*n* = 171)	Open RPS (*n* = 228)	*p* value	Laparoscopic RPS (*n* = 150)	Open RPS (*n* = 150)	*p* value	SMD
*Age*, *< 75 years,* *n (%)*^*a*^	135 (78.9)	175 (76.8)	0.60	114 (76.0)	117 (78.0)	0.690	0.047
Age (years)^b^	64 (13.3)	65 (13.1)	0.82	64.5 (13.8)	64.2 (13.5)	0.801	
*Sex*, *male*^*a*^	87 (50.9)	142 (62.3)	0.02	87 (58.0)	85 (56.7)	0.856	0.027
BMI (kg/m^2^)^b^	27.5 (4.8)	26.3 (4.9)	0.06	27.5 (4.8)	26.6 (4.8)	0.179	
*ASA classification*^*a*^			0.07			0.473	0.086
1–2	144 (84.2)	175 (76.8)		123 (82.0)	118 (78.7)		
3–4	27 (15.8)	53 (23.2)		27 (18.0)	32 (21.3)		
*Previous abdominal surgery, yes*^*a*^	92 (53.8)	113 (49.6)	0.40	74 (49.3)	71 (47.3)	0.690	0.040
*Previous abdominal surgery*			0.11			0.076	
No	79 (46.2)	115 (57.9)		76 (50.7)	79 (52.7)		
Yes, only laparoscopic	43 (25.1)	68 (29.8)		32 (21.3)	46 (30.7)		
Yes, open surgery	49 (28.7)	45 (19.7)		42 (28.0)	25 (16.7)		
*Previous liver surgery, yes*^*a*^	12 (7.0)	16 (7.0)	0.99	11 (7.3)	10 (6.7)	1.00	0.025
*Neoadjuvant chemotherapy, yes*^*a*^	53 (34.0)	81 (38.0)	0.52	47 (31.3)	47 (31.3)	1.00	0.0
*Diagnosis*^*a*^			0.61			0.344	0.082
Malignancy	151 (88.3)	205 (89.9)		132 (88.0)	136 (90.7)		
Diagnosis			0.67			0.357	
Colorectal liver metastasis	67 (39.2)	103 (45.2)		55 (36.7)	63 (42.0)		
Hepatocellular carcinoma	56 (32.7)	70 (30.7)		51 (34.0)	51 (34.0)		
Other malignancy	28 (16.4)	32 (14.0)		26 (17.3)	22 (14.7)		
Benign	20 (11.7)	23 (10.1)		18 (12.0)	14 (9.3)		
Cirrhosis^*a*^, yes	29 (17.0)	33 (14.5)	0.50	24 (16.0)	25 (16.7)	1.00	0.018
*Number of lesions*^*a*^			0.004			0.815	0.030
One	128 (74.9)	139 (61.0)		107 (71.3)	109 (72.7)		
Multiple	43 (25.1)	89 (39.0)		43 (28.7)	41 (27.3)		

Values in parentheses are percentages unless indicated otherwise

*ASA* American Society of Anesthesiologists, *BMI* Body Mass Index, *RPS* open right posterior sectionectomy, *PS* propensity score, *SMD* standardized mean difference

^a^Variables used in matching

^b^Mean (s.d.)

**Fig. 1:**
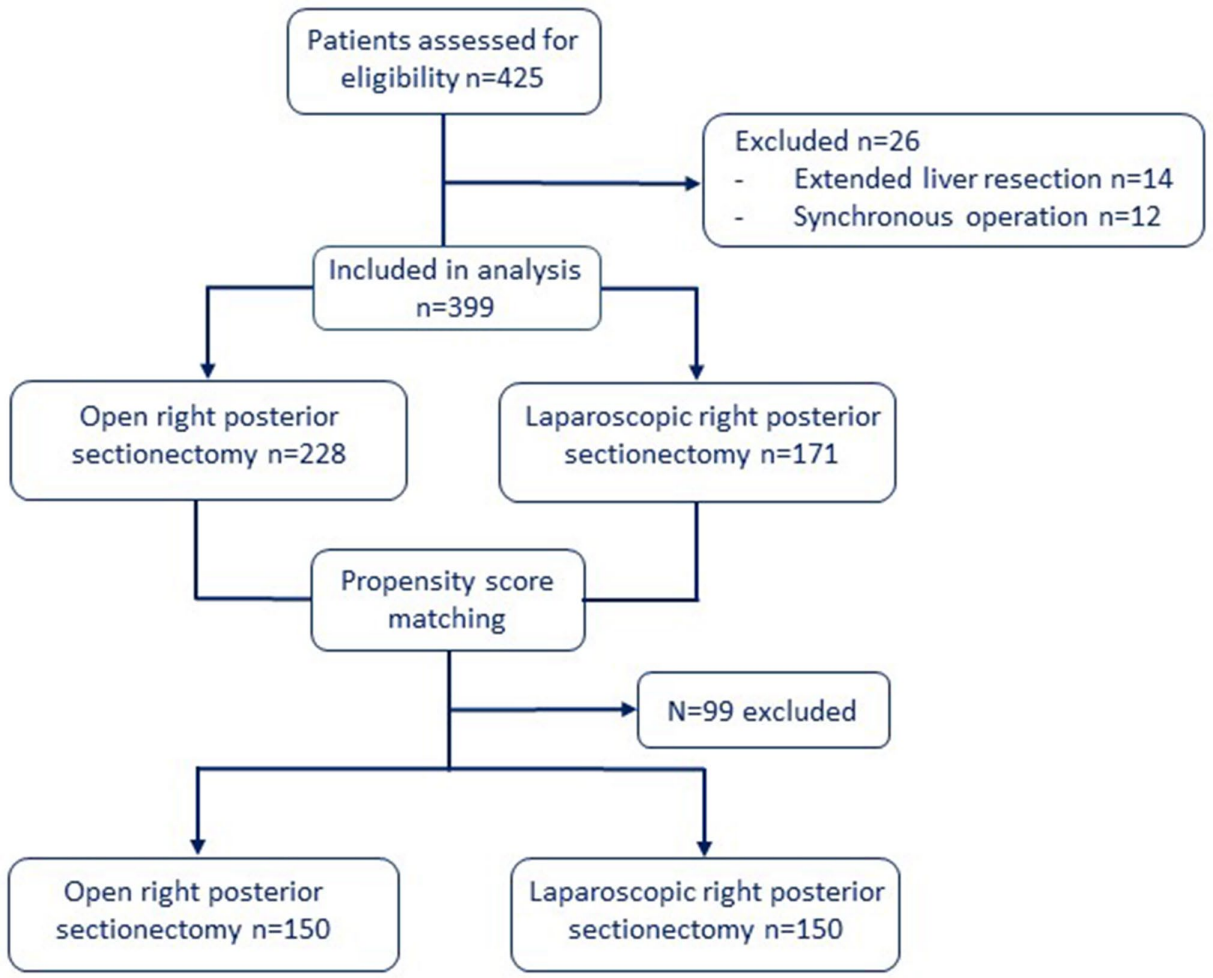
Flow chart of included patients

After propensity score matching, 150 patients remained in each group, a flowchart of the included patients is shown in Fig. 1. Matching was well balanced since all standardized mean differences for each variable used in matching were less than 0.1 (Table 1).

### Perioperative outcomes

Perioperative outcomes are provided in Table 2. The median operation duration was 12 minutes shorter in the LRPS group as compared to the ORPS group respectively [235 (195–285) vs. 247 min (195–315), *p* = 0.004]. The laparoscopic approach was associated with 180 mL less blood loss [median 260 vs 440 mL, *p* = 0.009]. Median length of stay was two days shorter in the LRPS group with five days (IQR 4–7) compared to seven days (IQR 6–10) in the ORPS group (*p* < 0.001). Conversion to an open approach was necessary in 21 patients (14%), in 10 patients due to bleeding, in seven patients due to concern for the oncological margin, in two patients there was technical inability to proceed laparoscopically and in two patients there were anesthetic problems. There were no intraoperative deaths recorded.

**Table 2:** Perioperative outcomes in the unmatched and matched cohort

	Before PS matching			After PS matching		
	Laparoscopic RPS (*n* = 171)	Open RPS (*n* = 228)	*p* value	Laparoscopic RPS (*n* = 150)	Open RPS (*n* = 150)	*p* value
Operative time in minutes^a^	235 (194–285)	243 (195–315)	0.043	235 (195–285)	247 (195–315)	0.004
Blood loss, (mL)^a^	250 (189–385)	440 (280–550)	< 0.001	260 (188–400)	440 (280–550)	0.009
Pringle’s manoeuver	128 (74.9)	135 (59.2)	0.001	112 (74.7)	94 (62.7)	0.033
Pringle (min)^b^	33 (20)	30 (19)	0.858	37 (17)	36 (15)	0.637
Conversion, *n* (%)	23 (13.5)	na	–	21 (14.0)	na	–
Length of stay (days)^a^	5 (4–7)	7 (6–10)	< 0.001	5 (4–7)	7 (6–10)	< 0.001
R0 resection	163 (95.3)	212 (93.0)	0.325	144 (96.0)	141 (94)	0.607
Number of lesions^a^	1 (1–1)	1 (1–2)	< 0.001	1 (1–2)	1 (1–2)	1.0
Size maximum lesion (mm)^b^	49 (27)	53 (29)	0.272	49 (26)	54 (28)	0.201
90-day mortality	0 (0)	2 (0.9)	0.199	0 (0)	1 (0.7)	1.0

Values in parentheses are percentages unless indicated otherwise

*RPS* open right posterior sectionectomy; *na* not applicable

^a^Values are median (IQR)

^b^Mean (SD)

### Pathology

The mean size of the largest lesion was 49 mm in LRPS patients and 54 mm in ORPS patients (*p* = 0.201). The R0 resection rate did not significantly differ between the two groups (Table 2).

### Complications

In the LRPS group 37 patients had one or more complications recorded versus 50 patients in the ORPS group, (*p* = 0.098), with 106 complications recorded in total (44 vs. 62 for laparoscopic and open respectively). An overview of the classification of complications and number of major complications is shown in Table 3. The rate of Accordion ≥ 3 complications (8 versus 9, *p* = 1.0) and the comprehensive complication index did not differ significantly between LRPS and ORPS respectively. There was one death recorded in the ORPS group as a result of bile leakage 56 days after surgery.

**Table 3:** Postoperative complications in the matched cohort

	Laparoscopic RPS (*n* = 150)	Open RPS (*n* = 150)	*p* value
*Accordion classification*			
Grade 1	7 (4.7)	9 (6.0)	
Grade 2	22 (14.7)	32 (21.3)	
Grade 3	7 (4.7)	6 (4.0)	
Grade 4	1 (0.7)	3 (2.0)	
Grade 5	0	0	
Grade 6	0	0	
Overall complications	37 (24.7)	50 (33.3)	0.098
Number of patients with one or more major complication^b^	8 (5.3)	9 (6.0)	1.0
Type of major complication^b^	9 complications	13 complications	
Bile leak	4	4	
Bleeding	1	3	
Intra-abdominal collection	1	3	
Ascitis	1	2	
Ileus	–	1	
Pulmonary infection	2	–	
Comprehensive complication index^a^	0 (0–20.9)	0 (0–2.2)	0.473

Values in parentheses are percentages unless indicated otherwise

*RPS* right posterior sectionectomy

^a^Values are median (IQR)

^b^Accordion ≥ 3

### Sensitivity analysis

Data of the sensitivity analysis excluding the first 10 LRPS per center are summarized in Table 4 and Table 5. In the unmatched cohort there were fewer patients in the LRPS group with ASA 3–4 [*n* = 11, 11.2% vs. *n* = 53, 23.3%, *p* = 0.012), a previous history of abdominal surgery (61.2% vs. 49.6%) and fewer with multiple lesions (20.4% vs. 39.0%). Matching was well balanced. In the matched cohort, operative time was shorter (median 225 vs. 269 min *p* = 0.013), blood loss less (median 230 vs. 420 mL, *p* < 0.001) and Pringle maneuver was done more often (84.9 vs. 66.3%, *p* = 0.008) in the LRPS group compared to the ORPS group respectively. Length of stay was shorter in the LRPS group (median 5 vs. 7 days, *p* < 0.001). There was no difference in Accordion-3 complications or 90-day mortality (Table 5).

**Table 4:** Sensitivity analysis perioperative outcomes before and after propensity score matching

	Before PS matching			After PS matching			
	Laparoscopic RPS (*n* = 98)	Open RPS (*n* = 228)	*p* value	Laparoscopic RPS (*n* = 86)	Open RPS (*n* = 86)	*p* value	SMD
Age, < 75 years, *n* (%)^a^	73 (74.5)	175 (76.8)	0.660	64 (74.4)	67 (77.9)	0.591	0.08
Age (years)^b^	68 (11)	65 (13)	0.065	68 (11)	65 (13)	0.066	
Sex, male^a^	53 (54.1)	142 (62.3)	0.166	50 (58.1)	49 (57.0)	1.00	0.02
BMI (kg/m^2^)^b^	27.1 (4.8)	26.3 (4.9)	0.652	27.2 (4.8)	26.2 (4.4)	0.100	
*ASA classification*^a^			0.012			1.00	0.03
1–2	87 (88.8)	175 (76.8)		75 (87.2)	76 (88.4)		
3–4	11 (11.2)	53 (23.2)		11 (12.8)	10 (11.6)		
*Previous abdominal surgery, yes*^a^	60 (61.2)	113 (49.6)	0.032	50 (58.1)	47 (54.7)	0.508	0.07
*Previous abdominal surgery*			0.053			0.244	
No	38 (38.8)	115 (50.4)		36 (41.9)	39 (45.3)		
Yes, only laparoscopic	28 (28.6)	68 (29.8)		26 (30.2)	32 (37.2)		
Yes, open surgery	32 (32.7)	45 (19.7)		24 (27.9)	15 (17.4)		
*Previous liver surgery, yes* ^a^	10 (10.2)	16 (7.0)	0.330	7 (8.1)	5 (5.8)	0.625	0.08
Neoadjuvant chemotherapy, yes	39 (76.5)	81 (78.6)	0.464	35 (40.7)	32 (37.2)	0.549	0.07
*Diagnosis*^*a*^			0.071			0.687	0.10
Malignancy	94 (95.9)	205 (89.9)		84 (97.7)	80 (93.0)		
Diagnosis			0.239			0.667	
Colorectal liver metastasis	50 (51.0)	103 (45.2)		43 (50.0)	41 (47.7)		
Hepatocellular carcinoma	27 (27.6)	70 (30.7)		26 (30.2)	26 (30.2)		
Other malignancy	17 (17.3)	32 (14.0)		13 (15.1)	13 (15.1)		
Benign	4 (4.1)	23 (10.1)		4 (4.7)	6 (7.0)		
Cirrhosis^a^, yes	12 (12.2)	33 (14.5)	0.593	11 (12.8)	7 (8.1)	0.289	0.13
*Number of lesions*^a^			0.001			0.250	0.08
One	78 (79.6)	139 (61.0)		67 (77.9)	70 (81.4)		
Multiple	20 (20.4)	89 (39.0)		19 (22.1)	16 (18.6)		

Values in parentheses are percentages unless indicated otherwise

*ASA* American Society of Anesthesiologists, *BMI* Body Mass Index, *RPS* right posterior sectionectomy, *PS* propensity score, *SMD* standardized mean difference

^a^Variables used in matching

^b^Mean (SD)

**Table 5:** Sensitivity analysis excluding first 10 laparoscopic right posterior sectionectomy patients per center and after propensity score matching

	Laparoscopic RPS (*n* = 86)	Open RPS (*n* = 86)	*p* value
Operative time in minutes^a^	225 (195–285)	269 (199–315)	0.013
Blood loss, (mL)^a^	230 (190–310)	420 (250–500)	< 0.001
Pringle’s manoeuver	73 (84.9)	57 (66.3)	0.008
Pringle (minutes)^b^	32 (13)	33 (17)	0.539
Conversion	10 (11.6)	NA	–
Length of stay (days)^a^	5 (4–6)	7 (6–9)	< 0.001
R0 resection	78 (90.7)	72 (83.7)	0.197
Number of lesions^a^	1 (1–1)	1 (1–1)	0.205
Size maximum lesion (mm)^b^	51 (24)	56 (28)	0.309
Accordion ≥ 3 complication	5 (5.8)	4 (4.7)	1.00
90-day mortality	0 (0)	0 (0)	1.00

Values in parentheses are percentages unless indicated otherwise

*RPS* right posterior sectionectomy, *NA* not applicable

^a^Values are median (IQR)

^b^Mean (SD)

### Multivariable regression analysis

For postoperative outcomes that were significantly different between the two approaches (blood loss, operative time and length of stay), a multivariable regression analysis was performed to determine risk factors for these outcomes (Supplemental tables A-C). In univariable analyses more blood loss was significantly related to the open approach (*p* = 0.002) and higher ASA classification (*p* = 0.001), malignancy as indication for surgery (*p* = 0.010), receiving neoadjuvant chemotherapy (*p* = 0.049) and undergoing Pringles’ maneuver (*p* = 0.004). In multiple regression analysis, blood loss was significantly associated with the open approach, higher ASA classification and malignancy as diagnosis (S table A).

Operative time remained significantly shorter for the laparoscopic approach (*p* = 0.005), when correcting for the other predictor variables age, ASA and diagnoses. In the univariate analysis older patients (≥ 75 years) tended to require a shorter operative time (*p* = 0.061) and this was significant (*p* = 0.017) after controlling for surgical approach, sex, ASA level and diagnosis (S table B).

In the univariable analysis hospital stay was significantly longer for patients undergoing the open surgical approach (*p* < 0.001), patients with ASA 3–4 (*p* = 0.032), patients having had previous abdominal surgery (*p* = 0.045) and patients treated for a malignancy. In the multivariable analysis including the five significant predictor variables from univariable analysis, only open surgical approach and malignancy as operation indication remained significantly contributing to a longer length of stay (S table C).

## Discussion

This international multicenter retrospective propensity score-matched study showed advantages of the laparoscopic approach over the open approach to right posterior sectionectomy in terms of operative time, blood loss, and length of hospital stay.

In this study, the shorter operative time in the LRPS group compares favorably to previous studies where the open approach was usually the least time demanding [19–21]. Although factors that could impact operation duration were included in matching (i.e., previous abdominal history, previous liver surgery and 1 or multiple lesions), there might be other impactful factors on the operation duration which were not collected. For example, laparoscopic procedures were possibly performed by more experienced surgeons. The median difference between the two groups in terms of operation time is 12 min. Although this is a statistically significant difference, it is not a clinically meaningful difference. Equally, this could be said about blood loss, where a median difference of 180 mL was seen in the open and laparoscopic group.

With the laparoscopic approach, no large subcostal incision is needed, making it easier for patients to start mobilizing immediately after surgery. Additionally, laparoscopic abdominal surgery is associated with better pain control [19, 22]. This both aids to the significantly shorter hospital stay that was found in the laparoscopic group.

There was a clear increase in number of laparoscopic resections in the second half of the inclusion period. Since none of the centers had performed a LRPS before the studies inclusion period, it is likely that surgeons were still in their learning curve. This might be a confounding factor, therefore a sensitivity analysis was done eliminating the first 10 LRPS of each center. This did not change the main perioperative outcomes. Multiple studies have assessed the learning curve in LLS [23–25]. Whereas for minor laparoscopic liver resections the learning curve was 22 procedures [23], for major hepatectomy this was 45–55 procedure [24, 25]. A recent study specifically assessing the learning curve for the posterosuperior segments concluded that the learning curve was estimated to be a total of 65 procedures for anatomical resections [26]. A factor that could shorten the learning curve is the use of specific training. Halls et al. assessed the impact of training in LLS by comparing outcomes of surgeons who were self-taught with surgeons who received specific training. Surgeons with specific training had similar short- and medium term outcomes after 46 procedures as self-taught surgeons after 150 procedures [27]. Most of the participating centers in this study had either received training or provided training for other centers. All centers were doing minor LLS years before they started with the LRPS and additionally these are all high-volume centers (> 20 laparoscopic liver procedures annually).

Hospital volume could be another confounding factor. For example, a multicenter study investigating the impact of hospital volume in minimally invasive hepatectomy found that resections of the posterosuperior segments had higher overall (30.4 vs 18.7%) and severe (9.9 vs 4.0%) morbidity rates in centers performing two or fewer minimally invasive liver procedures/month, than in those undertaking a larger number [28]. Interestingly, a recent study investigating the effect of volume on postoperative morbidity and mortality in liver surgery included only centers performing > 20 procedures annually. They concluded there was no association between hospital volume and postoperative outcomes when looking only at high-volume centers (≥ 20 cases annually) [29]. Since all centers in the current study are considered high-volume, they should be comparable in that aspect.

Parenchymal-sparing liver surgery can be technically more challenging than larger resections. Although the learning curve for the posterior segments may be longer than for right hemi-hepatectomy, parenchymal-sparing liver surgery preserves more parenchyma without compromising short term outcomes [30–32]. Several studies have also reported that number of R0 resections is also not inferior when performing parenchymal-sparing resections [33–35]. Parenchymal-sparing resections may improve survival compared to anatomical resections for colorectal liver metastases [35, 36]. Several recent studies have reported good outcomes of minimally invasive parenchymal-sparing liver resections but randomized studies are lacking [37–39]

The current study only included laparoscopic and open right posterior sectionectomies. No patients undergoing a robotic procedure were included. A meta-analysis on robot-assisted liver surgery showed that this approach seems feasible for major liver resections; however, all included studies were retrospective and observational in nature [40]. Future studies on RPS should consider including the robotic approach as well.

Propensity score matching has been increasingly popular in recent years. In a systematic review of all surgical oncology studies published between 2002 and 2014 in the 15 highest impact surgical journals, the number of studies using PSM increased from 3–5 per year from 2005–2009 to 98 in 2014 [41]. PSM is reliable and provides excellent covariate balance in most circumstances [42]. A disadvantage of PSM, however, is the loss of data that occurs after matching [43]. By performing regression analyses it was not only possible to assess other risk factors for certain outcomes but additionally, data of the entire cohort could be used.

This study should be interpreted in the light of the following limitations. First, this is a retrospective study and treatment allocation bias was present with more patients with smaller tumors and a lower ASA classification in the laparoscopic group. However, propensity score matching was used to minimize the influence of bias by indication. With propensity score matching there is always a possibility of residual confounding, such as training levels of surgeons. No information was available about the exact experience of each surgeon performing the procedure. Furthermore, we did not compare long-term survival outcomes between LRPS and ORPS, which was mainly because this study included both benign and malignant conditions and because most LRPS were performed very recently (in 2017–2018), so long-term follow-up data were not available yet. These limitations highlight the need for more prospective multicenter studies to further investigate the possible advantages of laparoscopy in major liver surgery.

## Conclusion

When performed in high-volume centers, laparoscopic right posterior sectionectomy is feasible and safe. Future studies should focus on long-term oncological outcomes when comparing the laparoscopic to the open approach.

## Electronic supplementary material

Below is the link to the electronic supplementary material.

Supplementary file1 (DOCX 30 kb)

## References

[1] Reich H, McGlynn F, DeCaprio J, Budin R (1991). Laparoscopic excision of benign liver lesions. Obstet Gynecol.

[2] Azagra JS, Goergen M, Gilbart E, Jacobs D (1996). Laparoscopic anatomical (hepatic) left lateral segmentectomy-technical aspects. Surg Endosc.

[3] Landi F, De' Angelis N, Scatton O, Vidal X, Ayav A, Muscari F, Dokmak S, Torzilli G, Demartines N, Soubrane O, Cherqui D, Hardwigsen J, Laurent A (2017). Short-term outcomes of laparoscopic vs. open liver resection for hepatocellular adenoma: a multicenter propensity score adjustment analysis by the AFC-HCA-2013 study group. Surg Endosc.

[4] Fretland AA, Dagenborg VJ, Bjornelv GMW, Kazaryan AM, Kristiansen R, Fagerland MW, Hausken J, Tonnessen TI, Abildgaard A, Barkhatov L, Yaqub S, Rosok BI, Bjornbeth BA, Andersen MH, Flatmark K, Aas E, Edwin B (2018). Laparoscopic versus open resection for colorectal liver metastases: the OSLO-COMET Randomized Controlled Trial. Ann Surg.

[5] Han HS, Shehta A, Ahn S, Yoon YS, Cho JY, Choi Y (2015). Laparoscopic versus open liver resection for hepatocellular carcinoma: case-matched study with propensity score matching. J Hepatol.

[6] Berardi G, Van Cleven S, Fretland AA, Barkhatov L, Halls M, Cipriani F, Aldrighetti L, Abu Hilal M, Edwin B, Troisi RI (2017). Evolution of laparoscopic liver surgery from innovation to implementation to mastery: perioperative and oncologic outcomes of 2,238 patients from 4 European Specialized Centers. J Am Coll Surg.

[7] Ban D, Tanabe M, Ito H, Otsuka Y, Nitta H, Abe Y, Hasegawa Y, Katagiri T, Takagi C, Itano O, Kaneko H, Wakabayashi G (2014). A novel difficulty scoring system for laparoscopic liver resection. J Hepatobiliary Pancreat Sci.

[8] Rhu J, Kim SJ, Choi GS, Kim JM, Joh JW, Kwon CHD (2018). Laparoscopic versus open right posterior sectionectomy for hepatocellular carcinoma in a high-volume center: a propensity score matched analysis. World J Surg.

[9] Cho JY, Han HS, Yoon YS, Choi Y, Lee W (2015). Outcomes of laparoscopic right posterior sectionectomy in patients with hepatocellular carcinoma in the era of laparoscopic surgery. Surgery.

[10] D’Hondt M, Ovaere S, Knol J, Vandeputte M, Parmentier I, De Meyere C, Vansteenkiste F, Besselink M, Pottel H, Verslype C (2018). Laparoscopic right posterior sectionectomy: single-center experience and technical aspects. Langenbeck's Archives of Surgery.

[11] Siddiqi NN, Abuawwad M, Halls M, Rawashdeh A, Giovinazzo F, Aljaiuossi A, Wicherts D, D'Hondt M, Hilal MA (2018). Laparoscopic right posterior sectionectomy (LRPS): surgical techniques and clinical outcomes. Surg Endosc.

[12] (2019) Health Research Authority. Research Tissue Banks and Research Databases

[13] von Elm E, Altman DG, Egger M, Pocock SJ, Gotzsche PC, Vandenbroucke JP, Initiative S (2014). The Strengthening the Reporting of Observational Studies in Epidemiology (STROBE) Statement: guidelines for reporting observational studies. Int J Surg.

[14] Strasberg SM (2005). Nomenclature of hepatic anatomy and resections: a review of the Brisbane 2000 system. J Hepatobiliary Pancreat Surg.

[15] Strasberg SM, Linehan DC, Hawkins WG (2009). The accordion severity grading system of surgical complications. Ann Surg.

[16] Slankamenac K, Graf R, Barkun J, Puhan MA, Clavien PA (2013). The comprehensive complication index: a novel continuous scale to measure surgical morbidity. Ann Surg.

[17] Pugh RN, Murray-Lyon IM, Dawson JL, Pietroni MC, Williams R (1973). Transection of the oesophagus for bleeding oesophageal varices. Br J Surg.

[18] Austin PC (2011). Optimal caliper widths for propensity-score matching when estimating differences in means and differences in proportions in observational studies. Pharm Stat.

[19] Tozzi F, Berardi G, Vierstraete M, Kasai M, de Carvalho LA, Vivarelli M, Montalti R, Troisi RI (2018). Laparoscopic versus open approach for formal right and left hepatectomy: a propensity score matching analysis. World J Surg.

[20] Kasai M, Cipriani F, Gayet B, Aldrighetti L, Ratti F, Sarmiento JM, Scatton O, Kim KH, Dagher I, Topal B, Primrose J, Nomi T, Fuks D, Abu Hilal M (2018). Laparoscopic versus open major hepatectomy: a systematic review and meta-analysis of individual patient data. Surgery.

[21] Nachmany I, Pencovich N, Zohar N, Ben-Yehuda A, Binyamin C, Goykhman Y, Lubezky N, Nakache R, Klausner JM (2015). Laparoscopic versus open liver resection for metastatic colorectal cancer. Eur J Surg Oncol.

[22] Tiefenthal M, Asklid D, Hjern F, Matthiessen P, Gustafsson UO (2016). Laparoscopic and open right-sided colonic resection in daily routine practice. A prospective multicentre study within an Enhanced Recovery After Surgery (ERAS) protocol. Colorectal Dis.

[23] Lin CW, Tsai TJ, Cheng TY, Wei HK, Hung CF, Chen YY, Chen CM (2016). The learning curve of laparoscopic liver resection after the Louisville statement 2008: will it be more effective and smooth?. Surg Endosc.

[24] Nomi T, Fuks D, Kawaguchi Y, Mal F, Nakajima Y, Gayet B (2015). Learning curve for laparoscopic major hepatectomy. Br J Surg.

[25] van der Poel MJ, Besselink MG, Cipriani F, Armstrong T, Takhar AS, van Dieren S, Primrose JN, Pearce NW, Abu Hilal M (2016). Outcome and learning curve in 159 consecutive patients undergoing total laparoscopic hemihepatectomy. JAMA Surg.

[26] Berardi G, Aghayan D, Fretland AA, Elberm H, Cipriani F, Spagnoli A, Montalti R, Ceelen WP, Aldrighetti L, Abu Hilal M, Edwin B, Troisi RI (2019). Multicentre analysis of the learning curve for laparoscopic liver resection of the posterosuperior segments. Br J Surg.

[27] Halls MC, Alseidi A, Berardi G, Cipriani F, Van der Poel M, Davila D, Ciria R, Besselink M, D'Hondt M, Dagher I, Alrdrighetti L, Troisi RI, Abu Hilal M (2019). A comparison of the learning curves of laparoscopic liver surgeons in differing stages of the IDEAL paradigm of surgical innovation: standing on the shoulders of pioneers. Ann Surg.

[28] Vigano L, Cimino M, Aldrighetti L, Ferrero A, Cillo U, Guglielmi A, Ettorre GM, Giuliante F, Dalla Valle R, Mazzaferro V, Jovine E, De Carlis L, Calise F, Torzilli G, Italian Group of Minimally Invasive Liver S (2020). Multicentre evaluation of case volume in minimally invasive hepatectomy. Br J Surg.

[29] Olthof PB, Elfrink AKE, Marra E, Belt EJT, van den Boezem PB, Bosscha K, Consten ECJ, den Dulk M, Gobardhan PD, Hagendoorn J, van Heek TNT, JNM IJ, Klaase JM, Kuhlmann KFD, Leclercq WKG, Liem MSL, Manusama ER, Marsman HA, Mieog JSD, Oosterling SJ, Patijn GA, Te Riele W, Swijnenburg RJ, Torrenga H, van Duijvendijk P, Vermaas M, Kok NFM, Grunhagen DJ, Dutch Hepato Biliary Audit G (2020) Volume-outcome relationship of liver surgery: a nationwide analysis. Br J Surg 10.1002/bjs.11586 PMC7384098 32207856

[30] Lalmahomed ZS, Ayez N, van der Pool AE, Verheij J, JN IJ, Verhoef C,  (2011). Anatomical versus nonanatomical resection of colorectal liver metastases: is there a difference in surgical and oncological outcome?. World J Surg.

[31] von Heesen M, Schuld J, Sperling J, Grunhage F, Lammert F, Richter S, Schilling MK, Kollmar O (2012). Parenchyma-preserving hepatic resection for colorectal liver metastases. Langenbecks Arch Surg.

[32] Donadon M, Cescon M, Cucchetti A, Cimino M, Costa G, Pesi B, Ercolani G, Pinna AD, Torzilli G (2018). Parenchymal-sparing surgery for the surgical treatment of multiple colorectal liver metastases is a safer approach than major hepatectomy not impairing patients' prognosis: a bi-institutional propensity score-matched analysis. Dig Surg.

[33] Finch RJ, Malik HZ, Hamady ZZ, Al-Mukhtar A, Adair R, Prasad KR, Lodge JP, Toogood GJ (2007). Effect of type of resection on outcome of hepatic resection for colorectal metastases. Br J Surg.

[34] Guzzetti E, Pulitano C, Catena M, Arru M, Ratti F, Finazzi R, Aldrighetti L, Ferla G (2008). Impact of type of liver resection on the outcome of colorectal liver metastases: a case-matched analysis. J Surg Oncol.

[35] Mise Y, Aloia TA, Brudvik KW, Schwarz L, Vauthey JN, Conrad C (2016). Parenchymal-sparing hepatectomy in colorectal liver metastasis improves salvageability and survival. Ann Surg.

[36] Okumura S, Tabchouri N, Leung U, Tinguely P, Louvet C, Beaussier M, Gayet B, Fuks D (2019). Laparoscopic parenchymal-sparing hepatectomy for multiple colorectal liver metastases improves outcomes and salvageability: a propensity score-matched analysis. Ann Surg Oncol.

[37] Conrad C, Ogiso S, Inoue Y, Shivathirthan N, Gayet B (2015). Laparoscopic parenchymal-sparing liver resection of lesions in the central segments: feasible, safe, and effective. Surg Endosc.

[38] D'Hondt M, Yoshihara E, Vansteenkiste F, Steelant PJ, Van Ooteghem B, Pottel H, Devriendt D, Van Rooy F (2016). Laparoscopic parenchymal preserving hepatic resections in semiprone position for tumors located in the posterosuperior segments. Langenbecks Arch Surg.

[39] Cipriani F, Shelat VG, Rawashdeh M, Francone E, Aldrighetti L, Takhar A, Armstrong T, Pearce NW, Abu Hilal M (2015). Laparoscopic parenchymal-sparing resections for nonperipheral liver lesions, the diamond technique: technical aspects, clinical outcomes, and oncologic efficiency. J Am Coll Surg.

[40] Nota CL, Rinkes IHB, Molenaar IQ, van Santvoort HC, Fong Y, Hagendoorn J (2016). Robot-assisted laparoscopic liver resection: a systematic review and pooled analysis of minor and major hepatectomies. HPB (Oxford).

[41] Yao XI, Wang X, Speicher PJ, Hwang ES, Cheng P, Harpole DH, Berry MF, Schrag D, Pang HH (2017) Reporting and Guidelines in Propensity Score Analysis: A Systematic Review of Cancer and Cancer Surgical Studies. J Natl Cancer Inst 109 10.1093/jnci/djw323 PMC6059208 28376195

[42] Deb S, Austin PC, Tu JV, Ko DT, Mazer CD, Kiss A, Fremes SE (2016). A review of propensity-score methods and their use in cardiovascular research. Can J Cardiol.

[43] Elze MC, Gregson J, Baber U, Williamson E, Sartori S, Mehran R, Nichols M, Stone GW, Pocock SJ (2017). Comparison of propensity score methods and covariate adjustment: evaluation in 4 cardiovascular studies. J Am Coll Cardiol.

