# Supplementary material for: Laparoscopic versus open right posterior sectionectomy: an international, multicenter, propensity score-matched evaluation
Source: Surg Endosc. 2020 Nov 2;35(11):6139–49. doi: 10.1007/s00464-020-08109-y (PMC8523385; doi:10.1007/s00464-020-08109-y)
Supplement: Supplementary file 1 — Supplementary file1 (DOCX 30 kb) [file 464_2020_8109_MOESM1_ESM.docx]

**Supplemental Table A. Linear regression analysis with blood loss as outcome variable, n=399.**

| **Variables** | **Univariate Beta [95% CI]** | **P value** | **Beta [95% CI]** | **P value** |
| --- | --- | --- | --- | --- |
| Open approach  Laparoscopic approach | Reference  -204.54 [-332.68 to -76.4] | 0.002 | Reference  -175.6 [-303.5 to -47.7] | 0.007 |
| Age, ≤75 years | Reference |  |  |  |
| >75 years | 33.96 [-119.73- 187.65] | 0.664 |  |  |
| Sex, female | Reference |  |  |  |
| Male | -25.5 [-156.52-105.61] | 0.703 |  |  |
| ASA, class 1-2 | Reference |  | Reference |  |
| class 3-4 | 257.0 [101.36-412.62] | 0.001 | 190.4 [35.2 – 345.6] | 0.016 |
| Previous abdominal surgery, no | Reference |  |  |  |
| Yes | 16.1 [-113.66-145.80] | 0.808 |  |  |
| Previous liver surgery, no | Reference |  | Reference |  |
| Yes | 177.8 [-71.66 – 427.24] | 0.162 | 182.7 [-58.9- 424.2] | 0.138 |
| Diagnosis, benign | Reference |  | Reference |  |
| Malignancy | 289.3 [69.97-508.64] | 0.010 | 266.9 [47.0 – 486.8] | 0.017 |
| Cirrhosis, no | Reference |  |  |  |
| Yes | -100.3 [-282.71- 82.17] | 0.280 |  |  |
| Neoadjuvant chemo, no | Reference |  | Reference |  |
| Yes | -137.7 [-274.56 to -0.77] | 0.049 | -86.4 [-223.7 – 50.9] | 0.247 |
| Single lesion | Reference |  |  |  |
| Multiple lesions | -32.6 [-170.20-104.997] | 0.642 |  |  |
| Pringle maneuver, no | Reference |  | Reference |  |
| Yes | -198.8 [-334.83 to—62.66] | 0.004 | -131 [-266.61 - 4.480] | 0.058 |

**Supplemental table B. Linear regression analysis with operative time as outcome variable, n=399.**

| **Variables** | **Univariate Beta [95% CI]** | **P value** | **Beta [95% CI]** | **P value** |
| --- | --- | --- | --- | --- |
| Open approach | Reference |  | Reference |  |
| Laparoscopic approach | -28.4 [-47.16 to -9.72] | 0.003 | -26.8 [-45.5 to -8.2] | 0.005 |
| Age, ≤75 years | Reference |  | Reference |  |
| >75 years | -21.23 [-43.45 – 0.99] | 0.061 | -27.0 [-49.2 to -4.8] | 0.017 |
| Sex, | Reference |  |  |  |
| Male | 10.39 [-8.55 - 29.32] | 0.282 |  |  |
| ASA, | Reference |  | Reference |  |
| class 3-4 | 20.77 [-2.79 - 44.33] | 0.084 | 21.5 [-2.0-44.9] | 0.072 |
| Previous abdominal surgery, no | Reference |  |  |  |
| Yes | -4.99 [-23.71 – 13.73] | 0.601 |  |  |
| Previous liver surgery, no | Reference |  |  |  |
| Yes | 10.09 [-27.46-47.64] | 0.598 |  |  |
| Diagnosis, benign | Reference |  | Reference |  |
| Malignancy | -25.31 [-55.76-5.13] | 0.103 | -29.4 [-59.6 – 0.8] | 0.056 |
| Cirrhosis, no | Reference |  |  |  |
| Yes | 4.23 [-21.56 – 30.02] | 0.747 |  |  |
| Neoadjuvant chemo, no | Reference |  |  |  |
| Yes | 4.08 [-15.67 - 23.84] | 0.685 |  |  |
| Single lesion | Reference |  |  |  |
| Multiple lesions | 7.80 [-12.09 – 27.69] | 0.441 |  |  |

**Supplemental table C. Linear regression analysis with hospital stay as outcome variable with regression coefficients depicted with 95% CI, n=399.**

| **Variables** | **Univariate Beta [95% CI]** | **P value** | **Beta [95% CI]** | **P value** |
| --- | --- | --- | --- | --- |
| Open approach | Reference |  | Reference |  |
| Laparoscopic | -2.74 [-4.06-1.42] | <0.001 | -2.64 [1.17-8.11] | <0.001 |
| Age, ≤75 years | Reference |  |  |  |
| >75 years | 0.29 [-1.31-1.88] | 0.726 |  |  |
| Sex, | Reference |  |  |  |
| Male | 0.38 [-0.97-1.73] | 0.579 |  |  |
| ASA, | Reference |  | Reference |  |
| class 3-4 | 0.11 [0.15-3.46] | 0.032 | 1.50 [-0.13-3.13] | 0.072 |
| Previous abdominal surgery, no | Reference |  | Reference |  |
| Yes | -1.36 [-2.69 to -0.03] | 0.045 | -0.820 [-2.22-0.58] | 0.251 |
| Previous liver surgery, no | Reference |  |  |  |
| Yes | -0.81 [-3.42 – 1.79] | 0.540 |  |  |
| Diagnosis, benign | Reference |  | Reference |  |
| Malignancy | 3.11 [0.99-5.24] | 0.004 | 2.84 [0.68-5.00] | 0.010 |
| Cirrhosis, no | Reference |  |  |  |
| Yes | 0.11 [-1.73-1.95] | 0.904 |  |  |
| Neoadjuvant chemo, no | Reference |  | Reference |  |
| Yes | -0.95 [-2.36-0.46] | 0.186 | -0.188 [-1.68-1.31] | 0.804 |
| Single lesion | Reference |  |  |  |
| Multiple lesions | 0.49 [-0.93-1.91] | 0.498 |  |  |
